# Supplementary material for: The Use of Pre-Endoscopic Metoclopramide Does Not Prevent the Need for Repeat Endoscopy: A U.S. Based Retrospective Cohort Study
Source: Life (Basel). 2024 Apr 19;14(4):526. doi: 10.3390/life14040526 (PMC11051147; doi:10.3390/life14040526)
Supplement: Supplementary file 1 [file life-14-00526-s001.zip › life-2919002-supplementary.pdf]

Supplementary Materials

Table S1: ICD-10 and CPT Codes Used in Methodology

• ***Patient Diagnoses***		
PUD	Chronic or unspecified peptic ulcer, site unspecified, with both hemorrhage and perforation	ICD-10 K27.6
	Peptic ulcer, site unspecified	ICD-10 K27
	Acute peptic ulcer, site unspecified, with hemorrhage	ICD-10 K27.0
	Chronic or unspecified peptic ulcer, site unspecified, with hemorrhage	ICD-10 K27.4
	Acute peptic ulcer, site unspecified, with perforation	ICD-10 K27.1
	Acute peptic ulcer, site unspecified, with both hemorrhage and perforation	ICD-10 K27.2
	Acute peptic ulcer, site unspecified, without hemorrhage or perforation	ICD-10 K27.3
	Chronic peptic ulcer, site unspecified, without hemorrhage or perforation	ICD-10 K27.7
	Chronic or unspecified peptic ulcer, site unspecified, with perforation	ICD-10 K27.5
UGIB	Gastrointestinal hemorrhage, unspecified	ICD-10 K92.2
EGD	Esophagogastroduodenoscopy, flexible, transoral; with injection sclerosis of esophageal/gastric varices	CPT 43243
	Esophagogastroduodenoscopy, flexible, transoral; diagnostic, including collection of specimen(s) by brushing or washing, when performed (separate procedure)	CPT 43235
	Esophagogastroduodenoscopy, flexible, transoral; with directed submucosal injection(s), any substance	CPT 43236
	Esophagogastroduodenoscopy, flexible, transoral; with biopsy, single or multiple	CPT 43239
• ***Outcomes***		
Transfusion	Transfusion, blood or blood components	CPT 36430

ICD-10: International Classification of Diseases 10th Revision; CPT: Current Procedural Terminology; PUD: Peptic Ulcer Disease; UGIB: Upper gastrointestinal bleed; EGD: Esophagogastroduodenoscopy;
